# Meta-analysis and trial sequential analysis of ezetimibe for coronary atherosclerotic plaque compositions

**DOI:** 10.3389/fphar.2023.1166762

**Published:** 2023-03-27

**Authors:** Bofeng Chai, Youlu Shen, Yuhong Li, Xiaoyu Wang

**Affiliations:** ^1^ Graduate School of Qinghai University, Xining, China; ^2^ Affiliated Hospital of Qinghai University, Xining, China; ^3^ The Third People’s Hospital of Tianshui, Tianshui, China

**Keywords:** ezetimibe, coronary atherosclerotic plaques, compositions, meta-analysis, trial sequential analysis

## Abstract

**Background:** Lipid aggregation, inflammatory cell infiltration, fibrous cap formation, and disruption are the major causes of atherosclerotic cardiovascular disease (ASCVD) and the pathologic features of atherosclerotic plaques. Although ezetimibe’s role in decreasing blood lipids is widely known, there are insufficient data to determine which part of the drug has an effect on atherosclerotic plaque compositions.

**Objective:** The study aimed to systematically evaluate the efficacy of ezetimibe for coronary atherosclerotic plaque compositions.

**Methods:** Two researchers independently searched the PubMed, Embase, Cochrane Library, and Web of Science databases for randomized controlled trials (RCTs) on the efficacy of ezetimibe for coronary atherosclerotic plaques from inception until 22 January 2023. The meta-analysis and trial sequential analysis (TSA) were performed using Stata 14.0 and TSA 0.9.5.10 Beta software, respectively.

**Results:** Four RCTs were finally included this study, which comprised 349 coronary artery disease patients. Meta-analysis findings showed that, compared with the control group, intervention measures could effectively reduce the fibro-fatty plaque (FFP) volume [WMD = −2.90, 95% CI (−4.79 and −1.00), and *p* = 0.003 < 0.05]; there were no significant difference in the reduction of fibrous plaque (FP) volume [WMD = −4.92, 95% CI (−11.57 and 1.74), and *p* = 0.15 > 0.05], necrotic core (NC) volume [WMD = −2.26, 95% CI (−6.99 and 2.46), and *p* = 0.35 > 0.05], and change dense calcification (change DC) volume [WMD = −0.07, 95% CI (−0.34 and 0.20), and *p* = 0.62 > 0.05] between the treatment group and the control group. TSA findings showed more studies are still required to confirm the efficacy of ezetimibe for FP and NC in the future.

**Conclusion:** Compared to the control group, ezetimibe significantly decreased FFP, but it had no statistically significant difference on FP, NC, or change DC. According to TSA, further research will be required to confirm the efficacy of ezetimibe for FP and NC in the future.

## 1 Introduction

Lipid aggregation, inflammatory cell infiltration, fibrous cap formation, and disruption are the major causes of atherosclerotic cardiovascular disease (ASCVD) and the pathological features of atherosclerotic plaques (Falk et al., 2013; Dawson et al., 2022). The cholesterol absorption inhibitor, ezetimibe, in combination with statins has been shown to significantly reduce low-density lipoprotein cholesterol (LDL-C) levels and improve outcomes in acute coronary syndromes in a large sample, double-blind, randomized controlled trial (Cannon et al., 2015). As compared to statin treatment alone, ezetimibe and statin combination significantly decreased coronary plaque volume, according to Ueda et al. (2017).

Although ezetimibe’s role in decreasing blood lipids is widely known and it is recommended in clinical guidelines (Grundy et al., 2019; Mach et al., 2020), there are insufficient data to determine which part of the drug has an effect on atherosclerotic plaque compositions. Multiple investigations on the efficacy of ezetimibe on coronary atherosclerotic plaque compositions have been conducted, although the findings are not entirely consistent. In order to give information for therapeutic practices, the aim of this study was to systematically evaluate the efficacy of ezetimibe for coronary atherosclerotic plaque compositions.

## 2 Materials and methods

The Preferred Reporting Items for Systematic Reviews and Meta-Analysis (PRISMA) criteria (Page et al., 2021) were followed for performing this meta-analysis, which was registered in the International Prospective Register of Systematic Reviews.

### 2.1 Inclusion and exclusion criteria

Inclusion criteria were as follows: 1) study type: RCTs in English language; 2) patients: patients who meet the diagnostic criteria for all types of coronary atherosclerotic heart disease and underwent intravascular ultrasound (IVUS) examinations, regardless of disease duration and severity, gender, age, and region; 3) intervention measures: the treatment group was treated with ezetimibe combined with statin or ezetimibe monotherapy, while the control group was treated with placebo, statin monotherapy, or blank control; and 4) endpoints: ① fibro-fatty plaque volume (FFP, mm³), ② fibrous plaque volume (FP, mm³), ③ necrotic core volume (NC, mm³), and ④ change dense calcification volume (change DC, mm³). Endpoints should be measured and calculated according to *American College of Cardiology Clinical Expert Consensus Document on Standards for Acquisition, Measurement and Reporting of Intravascular Ultrasound Studies* (Mintz, 2001)*.*

Exclusion criteria were as follows: 1) patients with other types of coronary artery disease, structural heart disease, heart failure, cardiomyopathy, connective tissue disease, and other influencing factors; 2) repeated literature; 3) errors or incomplete study data; and 4) case reports, conference reports, experts’ experience, animal experiments, and reviews.

### 2.2 Literature search strategy, data extraction, and quality evaluation

Two investigators separately searched the PubMed, Embase, Cochrane Library, and the Web of Science databases from inception until 22 January 2023. The terms searched included “Coronary Vessels,” “Coronary Artery,” “Plaque, Atherosclerotic,” “Atherosclerotic Plaques,” “Ezetimibe,” and “randomized controlled trial.” The detailed search strategy is shown in Supplementary Material S1.

Two investigators conducted literature screening and full-text reading and then extracted the information required for this study independently.

According to the RCT risk of the bias assessment tool in the Cochrane Handbook for Systematic Reviews, two researchers assessed the methodological quality of the included literature from seven aspects: sequence generation, allocation concealment, blinding of participants and personnel, blinding of the outcome assessment, incomplete outcome data, selective outcome reporting, and other sources of bias. Each aspect was rated as “low risk,” “unclear risk,” or “high risk” based on the tool.

### 2.3 Statistical analysis

Statistical analysis was performed using Stata 14.0 (Stata Corporation, College Station, TX, USA) software. Continuous variables were analyzed using the weighted mean difference (WMD) as the pooled statistic, which described the 95% confidence interval (CI). Heterogeneity size was evaluated by the *I* ^2^ value and *p*-value; if the inter-study statistical heterogeneity was less (*I* ^2^ ≤ 50% or *p* ≥ 0.1), the fixed-effect model was used; if the inter-study heterogeneity was significant (*I* ^2^ > 50% or *p* < 0.1), the random effect model was used. To check if there was publication bias, Egger’s test was used and a funnel plot was generated. *p* < 0.05 was considered statistically significant for the pooled effect. Finally, the TSA 0.9.5.10 Beta software was used to perform TSA analysis on the associated results.

## 3 Results

### 3.1 Search results, basic characteristics, and quality evaluation results of the included literature

**FIGURE 1:**
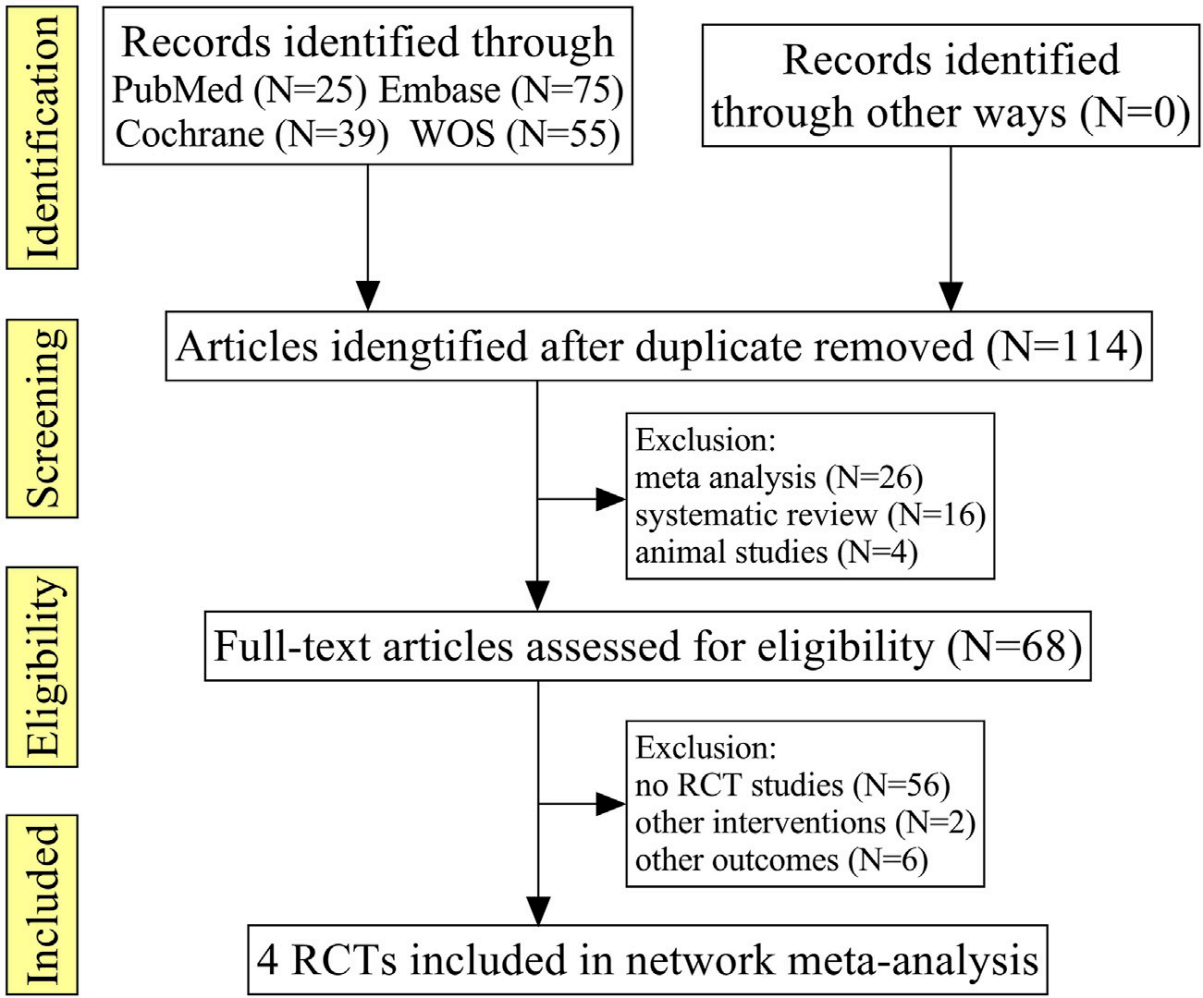
PRISMA flowchart with details of the literature search and study selection.

A total of 194 relevant literature reports were obtained in the initial screening, after excluding the repeated literature, meta-analyses, reviews, animal tests, no RCTs, or no match research contents, and four RCTs were finally included (Tomas et al., 2012; Jung et al., 2016; Mikkel et al., 2017; Kiyoshi et al., 2018), which comprised 349 patients; the literature screening processes are shown in Figure 1.

A multi-center research study was among the included research studies, which were primarily from Europe and Asia. The duration ranged from 3 to 12 months, and three of the studies were followed up for more than 10 months; the other basic characteristics are shown in Table 1.

**TABLE 1:** Basic information for the included studies.

Number	Study (year)	Region/country	Sample size	Male/female	Age	Intervention		Duration	Endpoint
			T/C	T; C	T/C	T (mg/d)	C (mg/d)		
1	Kiyoshi H (2018)	Japan multicenter	50/53	41/9; 41/12	63 ± 10/63 ± 12	EZ (10) + PI (2)	PI (2)	10 months	①②④
2	Mikkel H (2016)	Denmark	43/44	39/4; 36/8	55.3 ± 11.0/57.2 ± 9.1	EZ (10) + AT (80)	PL (10) + AT (80)	12 months	①②③④
3	Jung-H L (2016)	Korea	34/36	27/7; 27/9	60.9 ± 10.9/59.3 ± 10.7	EZ (10) + SI (40)	PR (20)	03 months	①②③④
4	Tomas K (2012)	Czech	42/47	33/9; 31/16	63.5 ± 9.3/65.1 ± 10.6	EZ (10) + AT (80)	AT (10)	12 months	①②③④

T, treatment group; C, control group; EZ, ezetimibe; PI, pitavastatin; AT, atorvastatin; PL, placebo; SI, simvastatin; PR, pravastatin. Endpoints: ① fibro-fatty plaque (FFP, mm^3^); ② fibrous plaque (FP, mm^3^); ③ necrotic core (NC, mm^3^); ④ change dense calcification (change DC, mm^3^).

**FIGURE 2:**
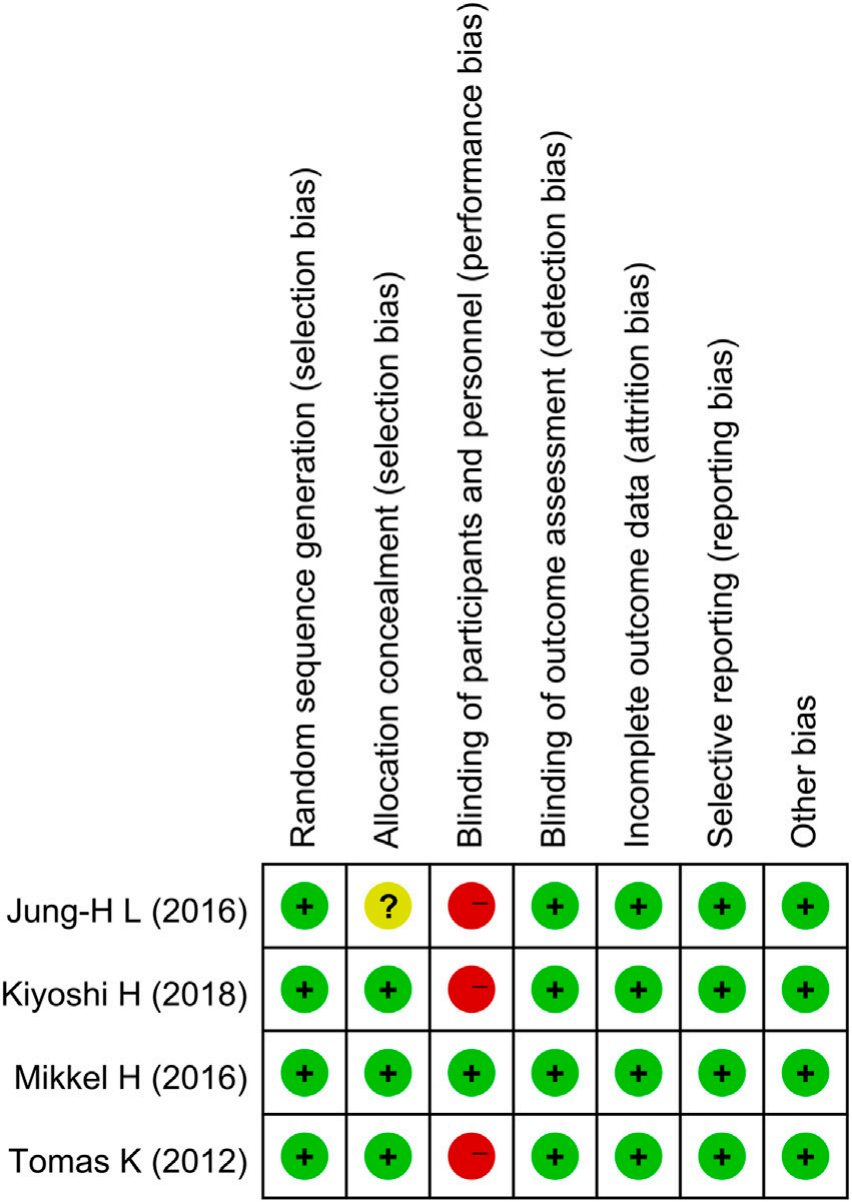
Risk of bias summary.

All four studies successfully described the random sequence generation method; however, one research’s allocation concealment was not well explained. Three studies successfully completed single blinding of the outcome assessment, while one study successfully completed the double-blinded study. The data on all studies were complete. All studies specifically described the interventions and outcome measures, as shown in Figure 2; Supplementary Material S2.

### 3.2 Meta-analysis

#### 3.2.1 Fibro-fatty plaque (FFP) volume

**FIGURE 3:**
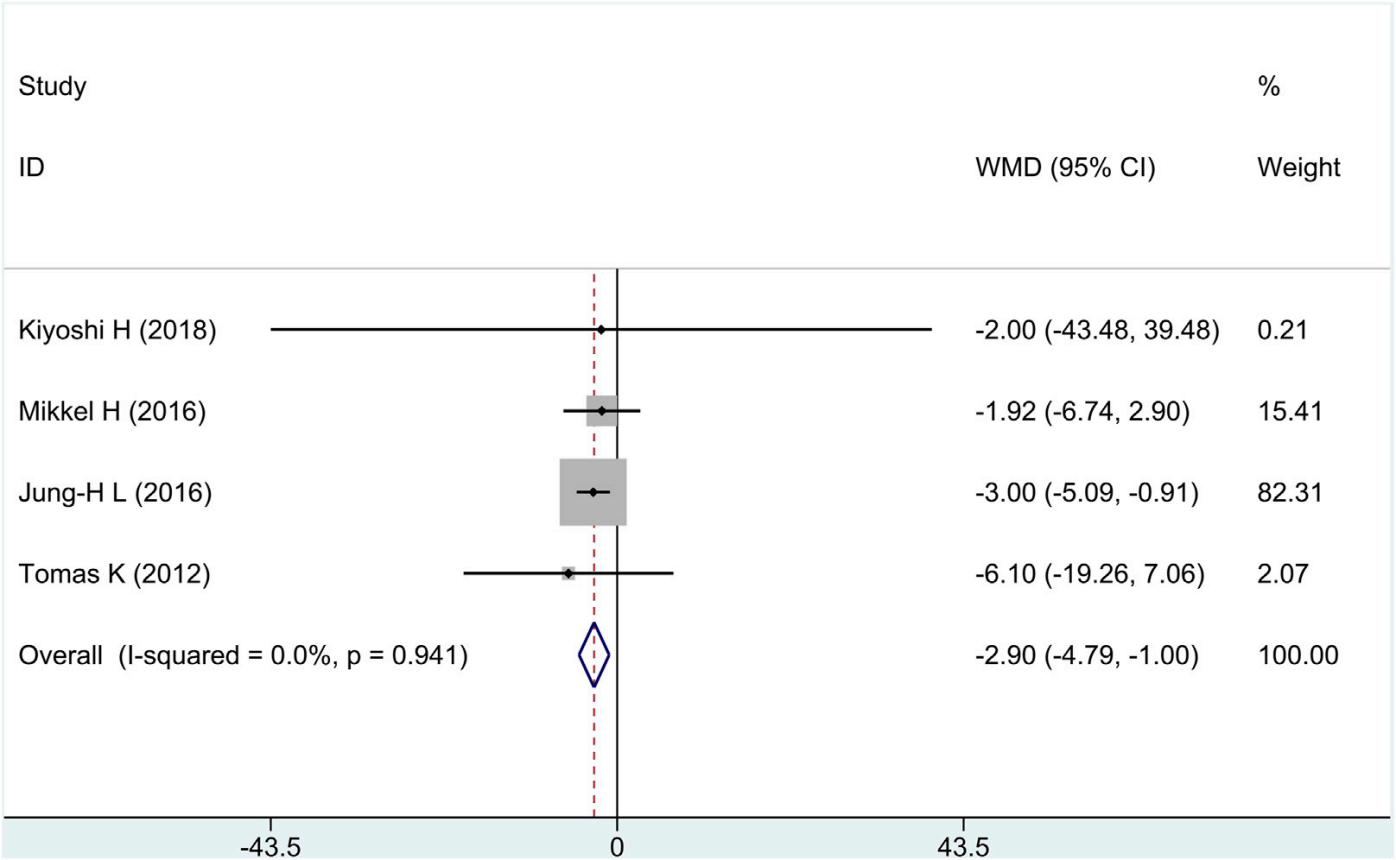
Forest plot for fibro-fatty plaque volume.

All research studies reported the efficacy of FFP, involving a total of 349 patients. There was no heterogeneity among the studies (*I* ^2^ = 0%, *p* = 0.94). Fixed-effects model analysis was carried out, and the result showed that compared with the control group, treatment group intervention measures could effectively reduce FFP, and there was a statistically significant difference [WMD = −2.90, 95% CI (−4.79 and −1.00), and *p* = 0.003], as shown in in Figure 3.

#### 3.2.2 Fibrous (FP) plaque volume

**FIGURE 4:**
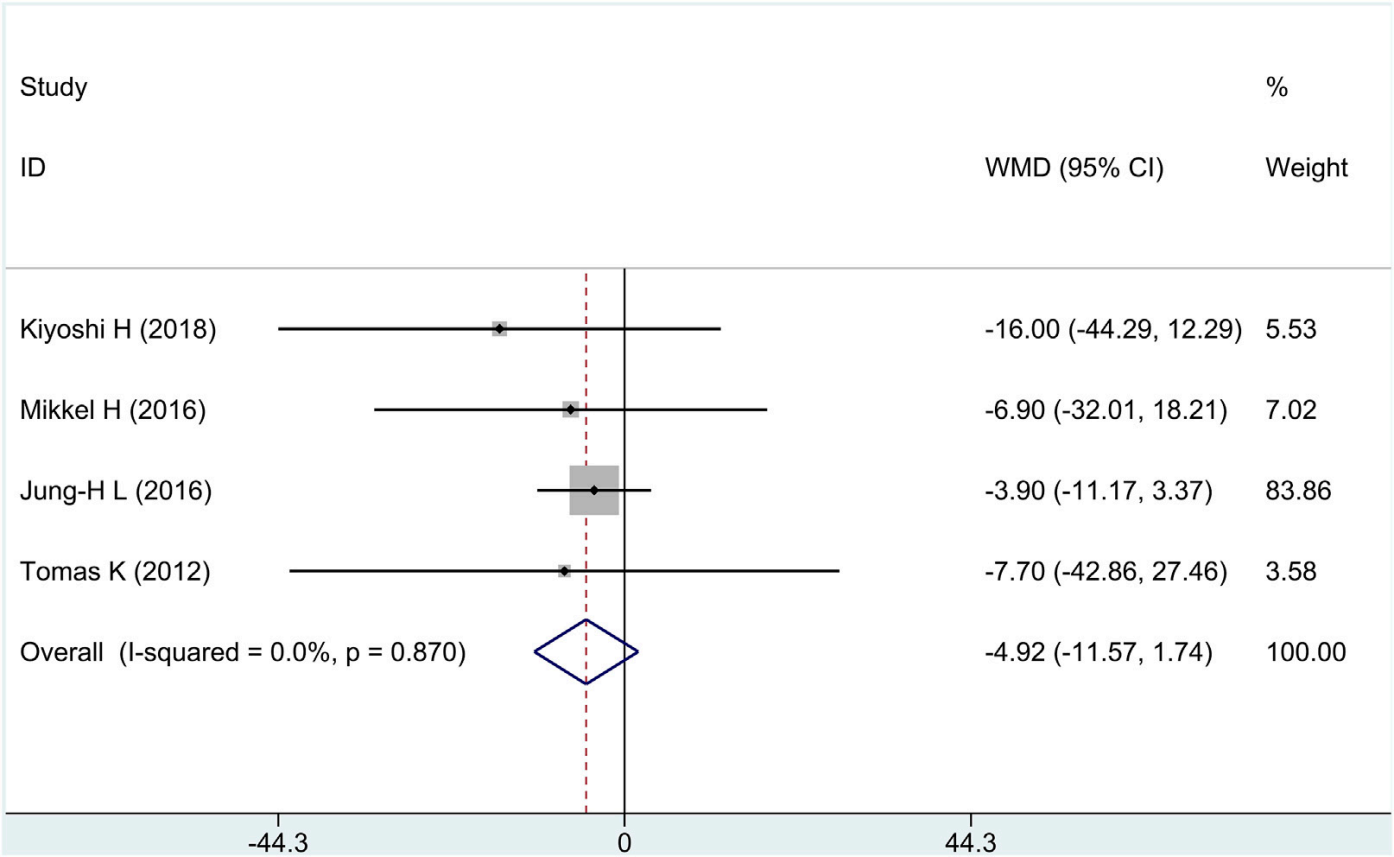
Forest plot for fibrous plaque volume.

All research studies reported the efficacy of FP, involving a total of 349 patients. There was no heterogeneity among the studies (*I* ^2^ = 0%, *p* = 0.87); fixed-effects model analysis was carried out, and the result showed that there was no significant difference in the reduction of FP between the treatment group and the control group [WMD = −4.92, 95% CI (−11.57 and 1.74), and *p* = 0.15], as shown in Figure 4.

#### 3.2.3 Necrotic core (NC) volume

**FIGURE 5:**
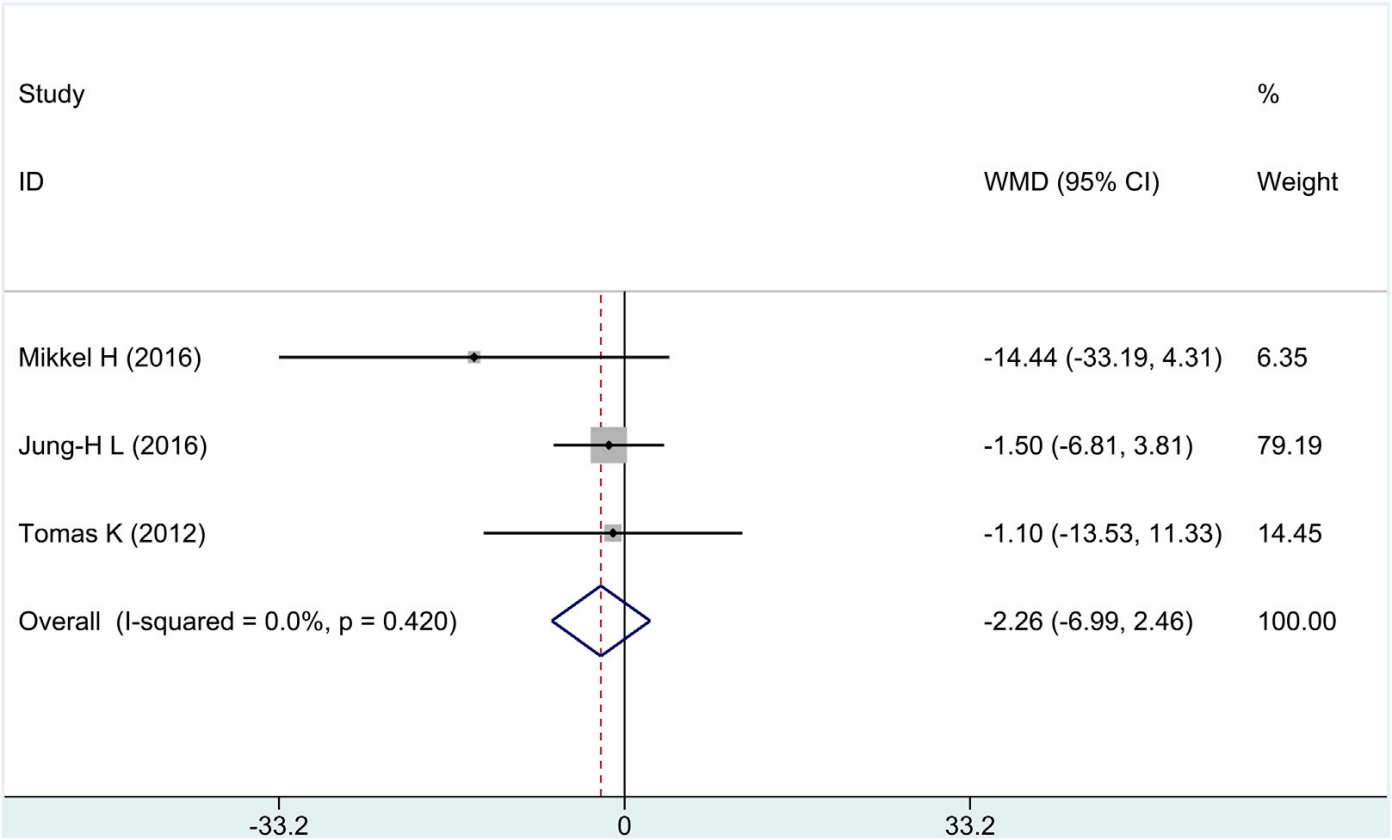
Forest plot for necrotic core volume.

Three of the four research studies reported the efficacy of NC, involving a total of 246 patients. There was no heterogeneity among the studies (*I* ^2^ = 0%, *p* = 0.42); fixed-effects model analysis was carried out, and the result showed that there was no significant difference in the reduction of NC between the treatment group and the control group [WMD = −2.26, 95% CI (−6.99 and 2.46), and *p* = 0.35], as shown in Figure 5.

#### 3.2.4 Change dense calcification (change DC) volume

**FIGURE 6:**
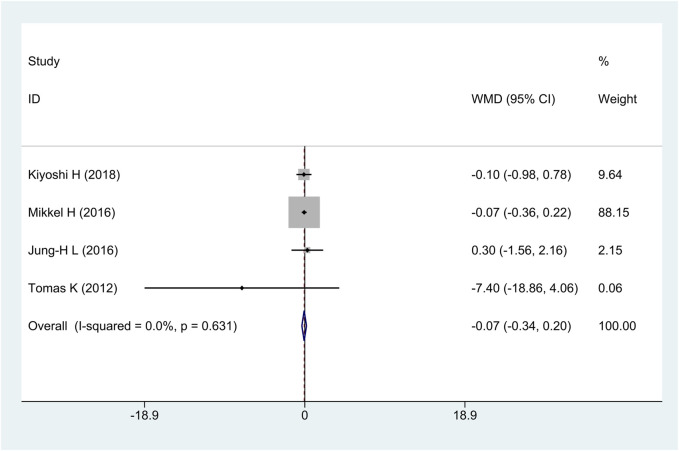
Forest plot for change dense calcification volume.

All research studies reported the efficacy of change DC, involving a total of 349 patients. There was no heterogeneity among the studies (*I* ^2^ = 0%, *p* = 0.63); fixed-effects model analysis was carried out, and the result showed that there was no significant difference in the reduction of change DC between the treatment group and the control group [WMD = −0.07, 95% CI (−0.34 and 0.20), and *p* = 0.62], as shown in Figure 6.

### 3.3 Subgroup analysis

We conducted subgroup analyses using patients’ continents as the covariable since Asian and European patients had different genetic variables. The result showed that compared with the control group, treatment group intervention measures could effectively reduce FFP in Asian patients; there was a statistically significant difference [WMD = −3.00, 95% CI (−5.08 and −0.91), and *p* = 0.005], but there was no statistically significant difference [WMD = −2.41, 95% CI (−6.94, 2.11), and *p* = 0.296] in European patients. Between the treatment group and the control group, there was no statistically significant difference in the reduction of FP, NC, and change DC in Asian or European patients, as shown in Table 2.

**TABLE 2:** Results of subgroup analysis.

Endpoint	Asia/Europe	Number of studies	Heterogeneity		Effect model	Meta-analysis result		
			*I* ^2^ value (%)	*p*-value		WMD (95%CI)	*Z*-value	*p*-value
①	Asia	2	0.00	= 0.96	Fixed	−3.00 (−5.08, −0.91)	2.82	= 0.005
	Europe	2	0.00	= 0.56	Fixed	−2.41 (−6.94, 2.11)	1.05	= 0.296
②	Asia	2	0.00	= 0.42	Fixed	−4.65 (−11.69, 2.39)	1.29	= 0.196
	Europe	2	0.00	= 0.97	Fixed	−7.17 (−27.61, 13.27)	0.69	= 0.492
③	Asia	1	—	—	—	—	—	—
	Europe	2	26.0	= 0.25	Fixed	−5.17 (−15.53, 5.19)	0.98	= 0.328
④	Asia	2	0.00	= 0.70	Fixed	−0.03 (−0.82, 0.77)	0.07	= 0.947
	Europe	2	36.3	= 0.21	Fixed	−0.07 (−0.37, 0.22)	0.50	= 0.615

Endpoints: ① fibro-fatty plaque (FFP, mm^3^); ② fibrous plaque (FP, mm^3^); ③ necrotic core (NC, mm^3^) ; ④ change dense calcification (change DC, mm^3^). WMD, weighted mean difference.

### 3.4 Publication bias

**FIGURE 7:**
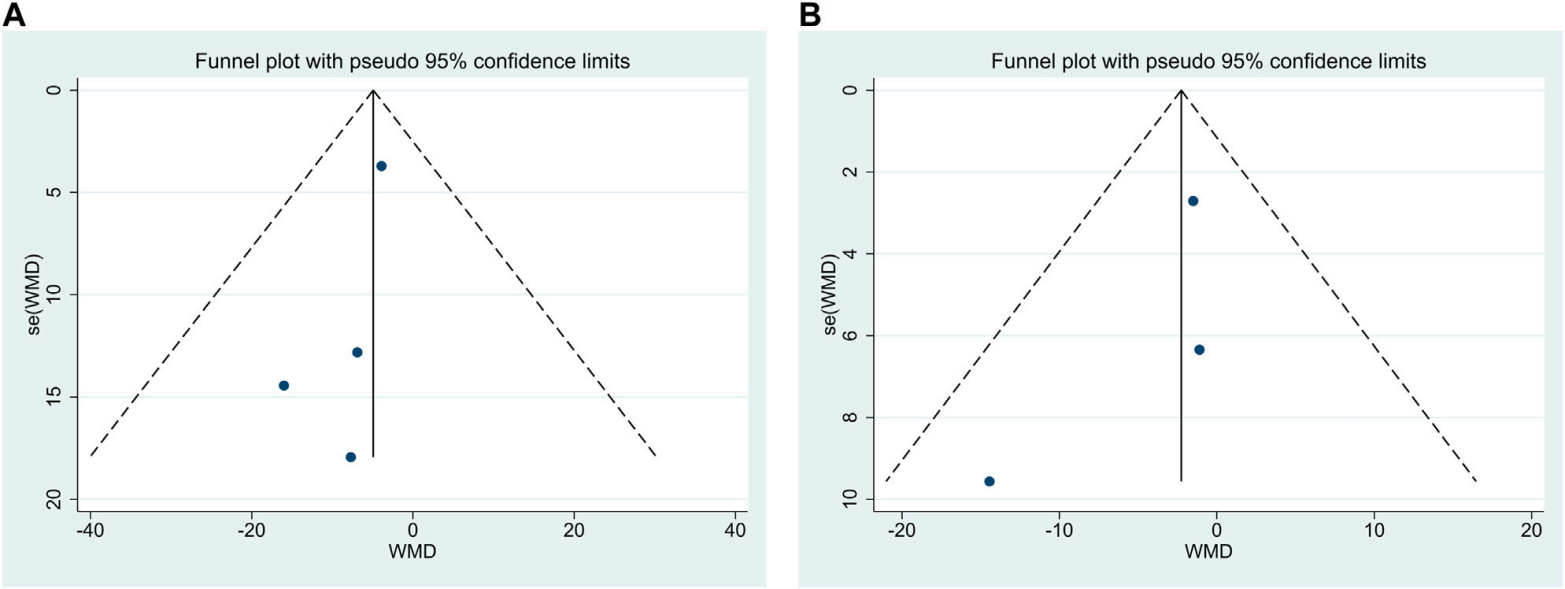
Funnel plots for fibrous plaque volume **(A)** and necrotic core volume **(B)**.

Publication bias was performed for FP and NC. The findings demonstrated that Egger’s test results were *p* = 0.20 > 0.05 and *p* = 0.47 > 0.05, respectively, and funnel plots were used, as shown in Figures 7A, B, indicating the little possibility of publication bias.

### 3.5 Trial sequential analysis

**FIGURE 8:**
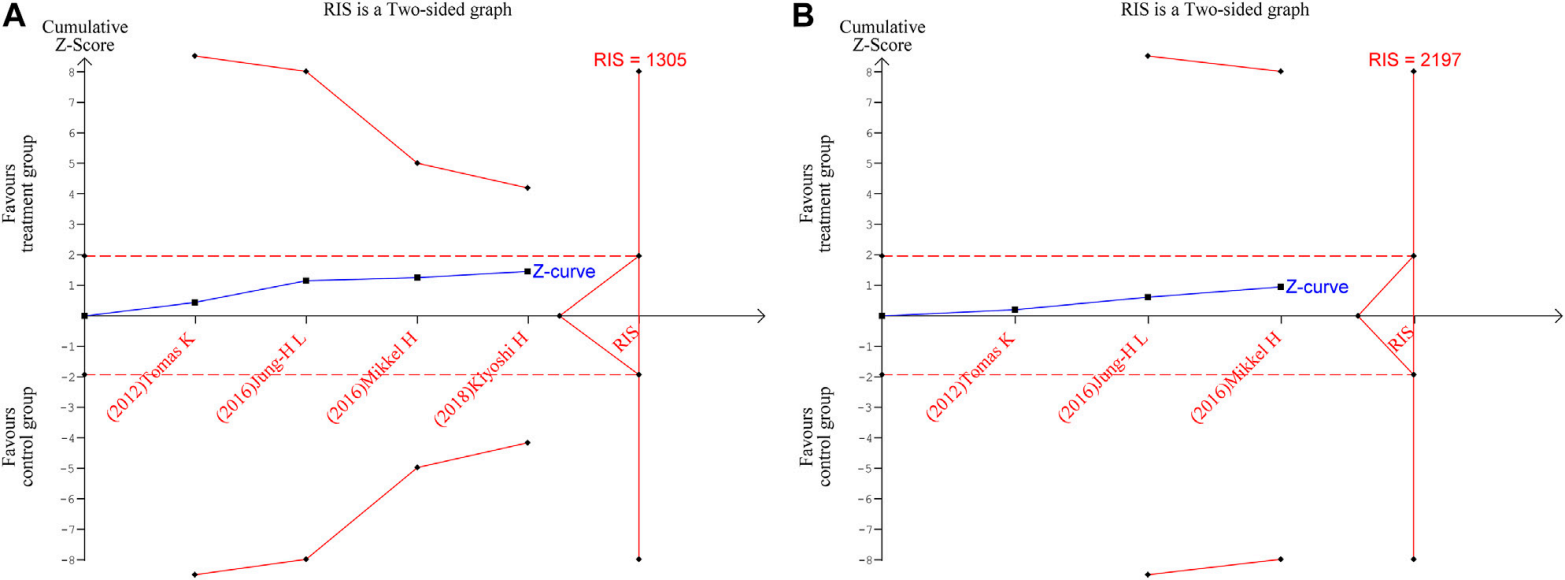
Trial sequential analysis for fibrous plaque volume **(A)** and necrotic core volume **(B)**.

TSA were performed for FP and NC. The parameters were set as follows: boundary type was two-side, Type 1 Error α = 5%, Type 2 Error β = 20%, and statistical power 1-β = 80%; the information axis was a sample size. The findings revealed that neither of the Z-curves crosses the traditional boundary values nor the TSA boundary values, and the accumulated information fell short of the required information size (RIS). Therefore, there was no statistically significant difference between the treatment group and the control group in terms of efficacy, and more studies are still required to confirm the efficacy in the future, as shown in Figures 8A, B.

## 4 Discussion

Since the endpoints were not published in the original publication as a mean ± standard deviation (mean ± SD), we converted data using the approach described by Luo et al. (2018). In terms of patients, acute coronary syndrome was studied in three trials, and stable angina, in one trial. According to the meta-analysis, patient differences did not cause heterogeneity among studies; compared to the control group, ezetimibe significantly decreased FFP, but it had no statistically significant difference on FP, NC, and change DC. FP and NC did not show any statistically significant differences between the treatment group and the control group, according to TSA, and further research will be required to confirm the efficacy in the future.

According to previous studies, the volume of the compositions in coronary atherosclerotic plaques can be impacted by statins. For example, statins increased FP and decreased FFP, but no change in NC was seen (Nasu et al., 2009). Ezetimibe inhibits the absorption of cholesterol from the intestine by blocking the Niemann–Pick-like 1 receptor. The findings of our meta-analyses demonstrated that ezetimibe combined with statins reduced FFP more significantly than statins alone; however, due to the limited sample size of the included trials, our study was inconclusive in terms of FP and NC, and TSA showed that further research is required. One of the included studies analyzed the whole investigated segment, and it is possible to infer that the changes in the worst segment are different from those in less affected parts of the vessel (Tomas et al., 2012). One study showed that in patients with ACS, additional reductions in LDL-C levels were associated with a reduction in cardiovascular events after ezetimibe combined with statin therapy compared with statins alone (Cannon et al., 2015). According to Newby (2005), both human and animal atherosclerotic plaques have higher levels of matrix metalloproteinase-9 (MMP-9), which can make plaque rupture more likely, resulting in thrombosis. According to Wang et al. (2016) findings, ezetimibe decreased the level of MMP-9. Additional research studies have demonstrated that a local high cholesterol concentration in foam cells can result in cholesterol crystal formation and localized inflammation (Duewell et al., 2010), inducing foam cell apoptosis and final formation of a lipid-rich necrotic core (Geng et al., 2003), while ezetimibe can reduce cholesterol crystal accumulation and plaque inflammation (Kataoka et al., 2015). These provide the basis for suggesting that ezetimibe may increase cardiovascular events due to plaque rupture and increase plaque stability.

According to Hong et al. (2009), statins had no significant effect on DC. The common occurrence of calcification in arteries, which mostly happens in the latter stages of atherosclerosis and has been linked to arterial degeneration and various metabolic diseases, has been thought of as a passive process (Otsuka et al., 2014; Jeevarethinam et al., 2017; Yahagi et al., 2017). According to a recent research study by Cho et al. (2018), both passive and active calcification processes are involved. We are unaware of any studies that demonstrate the ability of ezetimibe to inhibit both passive and active calcification processes, which may account for the lack of a statistically significant difference between the treatment group and the control group in terms of the change dense calcification volume. A study of 101 patients with coronary heart disease (CHD) by the Second Affiliated Hospital of Kunming Medical University (Qi et al., 2021) showed that the smaller the curvature and shallower the depth of dense calcification, the more prone they were to instability and rupture; curvature and depth are independent predictors of plaque rupture in patients with CHD. Future research studies on the effectiveness of ezetimibe on the curvature and depth of dense calcification might be carried out even though this study demonstrated that treatment measures in the treatment group and the control group had no statistical significance in the change dense calcification volume.

Lipid-lowering treatment is a first-line therapy for ACS; all of the included trials showed that the treatment and control groups can decrease total cholesterol (TC) and low-density lipoprotein (LDL) (Tomas et al., 2012; Jung et al., 2016; Mikkel et al., 2017; Kiyoshi et al., 2018), while only one study reported that the treatment group can increase high-density lipoprotein (HDL) (Kiyoshi et al., 2018).

## 5 Innovations and limitations

Innovations: As is known, this study is the first meta-analysis and trial sequential analysis to evaluate the efficacy of ezetimibe on the volume of each coronary atherosclerotic plaque composition.

Limitations: Patients with acute coronary syndrome and stable angina made up the majority of the study population, and the pathophysiological mechanisms of the former and the latter were not completely consistent. There may have been some deviation in data conversion because the data from several studies were not presented as the mean ± SD. Statins and their dose of four RCTs were different. A few inconclusive results show up as results of the limited sample size and the overall amount of literature; more high-quality randomized controlled trials can be conducted in the future.

## 6 Conclusion

This meta-analysis demonstrated that, compared to the control group, ezetimibe significantly decreased FFP, but it had no statistically significant difference on FP and NC; trial sequential analysis also showed that the meta-analysis results were inconclusive, and further research will be required to confirm the efficacy in the future. This meta-analysis showed that, compared to the control group, ezetimibe also had no statistically significant difference on change DC.

## References

[B1] Cannon C. P. Blazing M. A. Giugliano R. P. McCagg A. White J. A. Theroux P. (2015). Ezetimibe added to statin therapy after acute coronary syndromes. N. Engl. J. Med. 372, 2387–2397. 10.1056/NEJMoa1410489 26039521

[B2] Cho K. I. Sakuma I. Sohn I. S. Jo S. H. Koh K. K. (2018). Inflammatory and metabolic mechanisms underlying the calcific aortic valve disease. Atherosclerosis 277, 60–65. 10.1016/j.atherosclerosis.2018.08.029 30173080

[B3] Dawson L. P. Lum M. Nerleker N. Nicholls S. J. Layland J. (2022). Coronary atherosclerotic plaque regression: Jacc state-of-the-art review. J. Am. Coll. Cardiol. 79, 66–82. 10.1016/j.jacc.2021.10.035 34991791

[B4] Duewell P. Kono H. Rayner K. J. Sirois C. M. Vladimer G. Bauernfeind F. G. (2010). Nlrp3 inflammasomes are required for atherogenesis and activated by cholesterol crystals. Nature 464, 1357–1361. 10.1038/nature08938 20428172 PMC2946640

[B5] Falk E. Nakano M. Bentzon J. F. Finn A. V. Virmani R. (2013). Update on acute coronary syndromes: The pathologists' view. Eur. Heart J. 34, 719–728. 10.1093/eurheartj/ehs411 23242196

[B6] Geng Y. J. Phillips J. E. Mason R. P. Casscells S. W. (2003). Cholesterol crystallization and macrophage apoptosis: Implication for atherosclerotic plaque instability and rupture. Biochem. Pharmacol. 66, 1485–1492. 10.1016/s0006-2952(03)00502-1 14555225

[B7] Grundy S. M. Stone N. J. Bailey A. L. Beam C. Birtcher K. K. Blumenthal R. S. (2019). 2018 AHA/ACC/AACVPR/AAPA/ABC/ACPM/ADA/AGS/APhA/ASPC/NLA/PCNA guideline on the management of blood cholesterol: A report of the American college of cardiology/American heart association task force on clinical practice guidelines. Circulation 139, e1082–e1143. 10.1161/CIR.0000000000000625 30586774 PMC7403606

[B8] Hong M. K. Park D. W. Lee C. W. Lee S. W. Kim Y. H. Kang D. H. (2009). Effects of statin treatments on coronary plaques assessed by volumetric virtual histology intravascular ultrasound analysis. Jacc Cardiovasc Interv. 2, 679–688. 10.1016/j.jcin.2009.03.015 19628193

[B9] Jeevarethinam A. Venuraju S. Dumo A. Ruano S. Mehta V. S. Rosenthal M. (2017). Relationship between carotid atherosclerosis and coronary artery calcification in asymptomatic diabetic patients: A prospective multicenter study. Clin. Cardiol. 40, 752–758. 10.1002/clc.22727 28543093 PMC6490331

[B10] Jung-H. L. Shin D. H. Kim B. K. Ko Y. G. Choi D. Jang Y. (2016). Early effects of intensive lipid-lowering treatment on plaque characteristics assessed by virtual histology intravascular ultrasound. Yonsei Med. J. 57, 1087–1094. 10.3349/ymj.2016.57.5.1087 27401638 PMC4960373

[B11] Kataoka Y. Puri R. Hammadah M. Duggal B. Uno K. Kapadia S. R. (2015). Cholesterol crystals associate with coronary plaque vulnerability *in vivo* . J. Am. Coll. Cardiol. 65, 630–632. 10.1016/j.jacc.2014.11.039 25677323

[B12] Kiyoshi H. Sonoda S. Kawasaki M. Otsuji Y. Murohara T. Ishii H. (2018). Effects of ezetimibe-statin combination therapy on coronary atherosclerosis in acute coronary syndrome. Circ. J. 82, 757–766. 10.1253/circj.CJ-17-0598 29212965

[B13] Luo D. Wan X. Liu J. Tong T. (2018). Optimally estimating the sample mean from the sample size, median, mid-range, and/or mid-quartile range. Stat. Methods Med. Res. 27, 1785–1805. 10.1177/0962280216669183 27683581

[B14] Mach F. Baigent C. Catapano A. L. Koskinas K. C. Casula M. Badimon L. (2020). 2019 esc/eas guidelines for the management of dyslipidaemias: Lipid modification to reduce cardiovascular risk. Eur. Heart J. 41, 111–188. 10.1093/eurheartj/ehz455 31504418

[B15] Mikkel H. Hansen H. S. Thayssen P. Antonsen L. Junker A. Veien K. (2017). Influence of ezetimibe in addition to high-dose atorvastatin therapy on plaque composition in patients with st-segment elevation myocardial infarction assessed by serial: Intravascular ultrasound with imap: The octivus trial. Cardiovasc. Revasc. Med. 18, 110–117. 10.1016/j.carrev.2016.11.010 27919638

[B16] Mintz G. (2001). American college of cardiology clinical expert consensus document on standards for acquisition, measurement and reporting of intravascular ultrasound studies (ivus). A report of the American college of cardiology task force on clinical expert consensus documents developed in collaboration with the European society of cardiology endorsed by the society of cardiac angiography and interventions. Eur. J. Echocardiogr. 2, 299–313. 10.1053/euje.2001.0133 11908481

[B17] Nasu K. Tsuchikane E. Katoh O. Tanaka N. Kimura M. Ehara M. (2009). Effect of fluvastatin on progression of coronary atherosclerotic plaque evaluated by virtual histology intravascular ultrasound. Jacc Cardiovasc Interv. 2, 689–696. 10.1016/j.jcin.2009.04.016 19628194

[B18] Newby A. C. (2005). Dual role of matrix metalloproteinases (matrixins) in intimal thickening and atherosclerotic plaque rupture. Physiol. Rev. 85, 1–31. 10.1152/physrev.00048.2003 15618476

[B19] Otsuka F. Sakakura K. Yahagi K. Joner M. Virmani R. (2014). Has our understanding of calcification in human coronary atherosclerosis progressed? Arterioscler. Thromb. Vasc. Biol. 34, 724–736. 10.1161/ATVBAHA.113.302642 24558104 PMC4095985

[B20] Page M. J. McKenzie J. E. Bossuyt P. M. Boutron I. Hoffmann T. C. Mulrow C. D. (2021). The prisma 2020 statement: An updated guideline for reporting systematic reviews. Syst. Rev. 10, 89. 10.1186/s13643-021-01626-4 33781348 PMC8008539

[B21] Qi Z. Jikun D. Fan Y. Zhi L. Hua G. Lin S. (2021). Relationship between spotty calcification and plaque stability in patients with coronary heart disease: an optical coherence tomography study. J. Clin. Cardiol. 37, 1117–1120. 10.13201/j.issn.1001-1439.2021.12.010

[B22] Tomas K. Mintz G. S. Skalicka H. Kral A. Horak J. Skulec R. (2012). Virtual histology evaluation of atherosclerosis regression during atorvastatin and ezetimibe administration: Heaven study. Circ. J. 76, 176–183. 10.1253/circj.cj-11-0730 22076422

[B23] Ueda Y. Hiro T. Hirayama A. Komatsu S. Matsuoka H. Takayama T. (2017). Effect of ezetimibe on stabilization and regression of intracoronary plaque - the zipangu study. Circ. J. 81, 1611–1619. 10.1253/circj.CJ-17-0193 28592751

[B24] Wang X. Zhao X. Li L. Yao H. Jiang Y. Zhang J. (2016). Effects of combination of ezetimibe and rosuvastatin on coronary artery plaque in patients with coronary heart disease. Heart Lung Circ. 25, 459–465. 10.1016/j.hlc.2015.10.012 26687339

[B25] Yahagi K. Kolodgie F. D. Lutter C. Mori H. Romero M. E. Finn A. V. (2017). Pathology of human coronary and carotid artery atherosclerosis and vascular calcification in diabetes mellitus. Arterioscler. Thromb. Vasc. Biol. 37, 191–204. 10.1161/ATVBAHA.116.306256 27908890 PMC5269516

