# Supplementary figures and images for: Meta-analysis and trial sequential analysis of ezetimibe for coronary atherosclerotic plaque compositions
Source: Front Pharmacol. 2023 Mar 27;14:1166762. doi: 10.3389/fphar.2023.1166762 (PMC10084938; doi:10.3389/fphar.2023.1166762)

**Supplementary Material S2**

Risk of bias graph


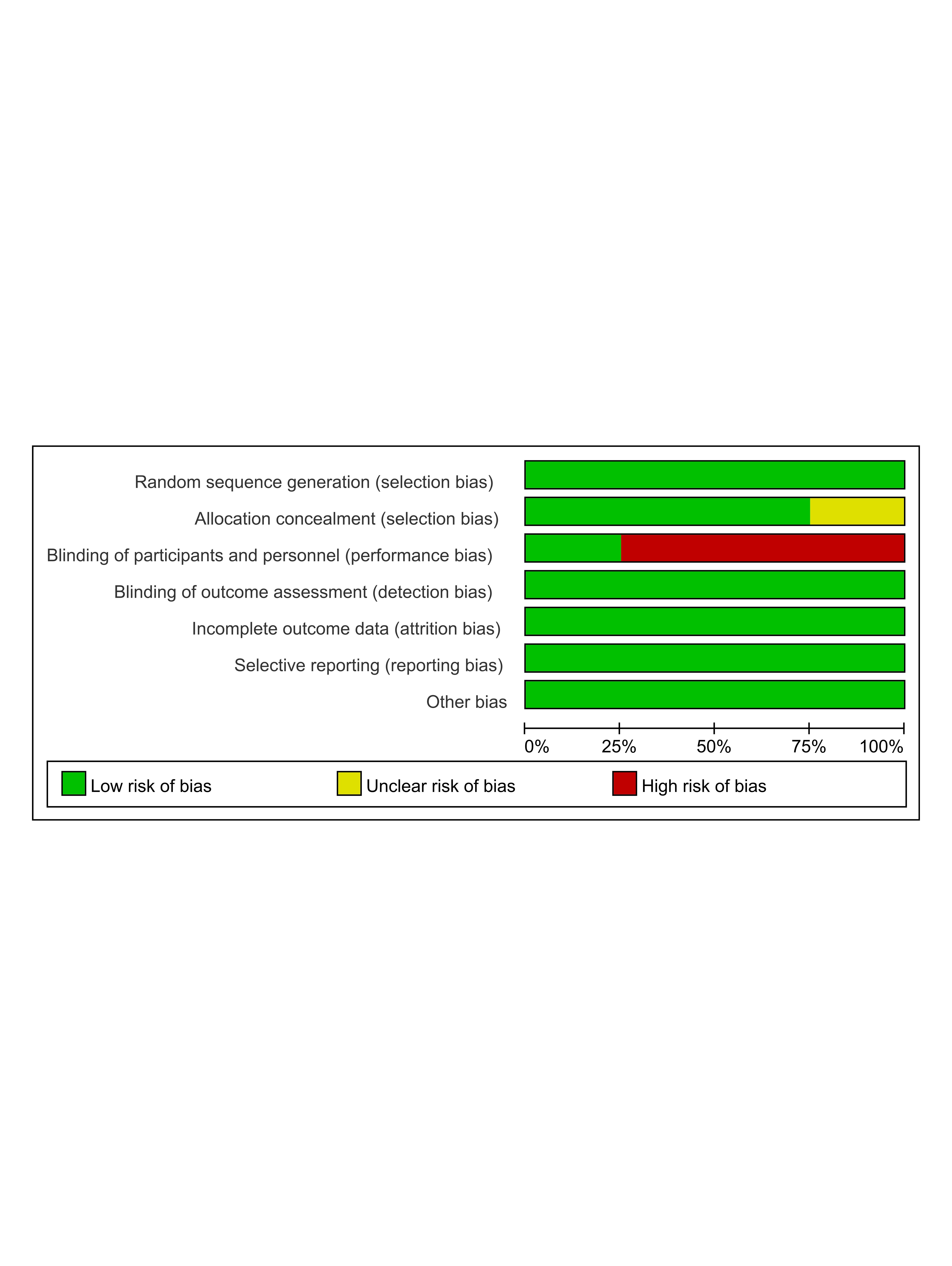

Supplement: Supplementary file 1 [file DataSheet2.docx]
