# Supplementary material for: Meta-analysis and trial sequential analysis of ezetimibe for coronary atherosclerotic plaque compositions
Source: Front Pharmacol. 2023 Mar 27;14:1166762. doi: 10.3389/fphar.2023.1166762 (PMC10084938; doi:10.3389/fphar.2023.1166762)
Supplement: Supplementary file 2 [file DataSheet1.docx]

**Supplementary Material S1**

**Search strategy of PubMed**

**#1 **"Coronary Vessels"[Mesh] Sort by: Most Recent****

****#2 (((((((coronary[Title/Abstract]) OR (Coronary Vessel[Title/Abstract])) OR (Vessel, Coronary[Title/Abstract])) OR (Vessels, Coronary[Title/Abstract])) OR (Coronary Arteries[Title/Abstract])) OR (Arteries, Coronary[Title/Abstract])) OR (Artery, Coronary[Title/Abstract])) OR (Coronary Artery[Title/Abstract])****

****#3 #1 or #2****

****#4 "Plaque, Atherosclerotic"[Mesh] Sort by: Most Recent****

****#5 (((((((((((((((Atherosclerotic Plaques[Title/Abstract]) OR (Atherosclerotic Plaque[Title/Abstract])) OR (Fatty Streak, Arterial[Title/Abstract])) OR (Arterial Fatty Streak[Title/Abstract])) OR (Arterial Fatty Streaks[Title/Abstract])) OR (Streak, Arterial Fatty[Title/Abstract])) OR (Fibroatheroma[Title/Abstract])) OR (Fibroatheromas[Title/Abstract])) OR (Fibroatheromatous Plaques[Title/Abstract])) OR (Fibroatheromatous Plaque[Title/Abstract])) OR (Plaque, Fibroatheromatous[Title/Abstract])) OR (Atheroma[Title/Abstract])) OR (Atheromas[Title/Abstract])) OR (Atheromatous Plaques[Title/Abstract])) OR (Atheromatous Plaque[Title/Abstract])) OR (Plaque, Atheromatous[Title/Abstract])****

****#6 #4 or #5****

****#7 "Ezetimibe"[Mesh] Sort by: Most Recent****

****#8 (((((((Ezetimib[Title/Abstract]) OR (Ezetrol[Title/Abstract])) OR (SCH 58235[Title/Abstract])) OR (58235, SCH[Title/Abstract])) OR (SCH-58235[Title/Abstract])) OR (SCH58235[Title/Abstract])) OR (Zetia[Title/Abstract])) OR ((1-(4-fluorophenyl[Title/Abstract])-(3R)-(3-(4-fluorophenyl)-(3S)-hydroxypropyl)-(4S)-(4-hydroxyphenyl)-2-azetidinone))****

****#9 #7 or #8****

****#10 ((((((((randomized controlled trial[Publication Type]) OR (randomized controlled study[Title/Abstract])) OR (controlled clinical trial[Title/Abstract])) OR (randomized[Title/Abstract])) OR (clinical trials as topic[Title/Abstract])) OR (Randomly[Title/Abstract])) OR (Trial[Title/Abstract])) OR (RCT[Title/Abstract])) OR (Random[Title/Abstract])****

****#11 #3 and #6 and #9 and #10****
